# Protein SIC Secreted from *Streptococcus pyogenes* Forms Complexes with Extracellular Histones That Boost Cytokine Production

**DOI:** 10.3389/fimmu.2018.00236

**Published:** 2018-02-22

**Authors:** Johannes Westman, Bhavya Chakrakodi, Johanna Snäll, Matthias Mörgelin, Martin Bruun Madsen, Ole Hyldegaard, Ariane Neumann, Inga-Maria Frick, Anna Norrby-Teglund, Lars Björck, Heiko Herwald

**Affiliations:** ^1^Program in Cell Biology, The Hospital for Sick Children, Toronto, ON, Canada; ^2^Department of Medicine, Karolinska Institutet, Center for Infectious Medicine, Karolinska University Hospital Huddinge, Stockholm, Sweden; ^3^Department of Clinical Sciences, Faculty of Medicine, Division of Infection Medicine, Lund University, Lund, Sweden; ^4^Rigshospitalet, Department of Intensive Care, Copenhagen University Hospital, Copenhagen, Denmark; ^5^Rigshospitalet, Hyperbaric Unit, Department of Anesthesia, Center of Head and Orthopedics, Copenhagen, Denmark

**Keywords:** extracellular histones, streptococcal inhibitor of complement, toll-like receptor, antimicrobial peptide, cytokines, *Streptococcus pyogenes*, innate immunity

## Abstract

Innate immunity relies on an effective recognition of the pathogenic microorganism as well as on endogenous danger signals. While bacteria in concert with their secreted virulence factors can cause a number of inflammatory reactions, danger signals released at the site of infection may in addition determine the amplitude of such responses and influence the outcome of the disease. Here, we report that protein SIC, Streptococcal Inhibitor of Complement, an abundant secreted protein from *Streptococcus pyogenes*, binds to extracellular histones, a group of danger signals released during necrotizing tissue damage. This interaction leads to the formation of large aggregates *in vitro*. Extracellular histones and SIC are abundantly expressed and seen colocalized in biopsies from patients with necrotizing soft-tissue infections caused by *S. pyogenes*. In addition, binding of SIC to histones neutralized their antimicrobial activity. Likewise, the ability of histones to induce hemolysis was inhibited in the presence of SIC. However, when added to whole blood, SIC was not able to block the pro-inflammatory effect of histones. Instead SIC boosted the histone-triggered release of a broad range of cytokines and chemokines, including IL-6, TNF-α, IL-8, IL-1β, IL-1ra, G-CSF, and IFN-γ. These results demonstrate that the interaction between SIC and histones has multiple effects on the host response to *S. pyogenes* infection.

## Introduction

*Streptococcus pyogenes* (group A *streptococcus*, GAS) is an important human pathogen that may cause a number of conditions from harmless throat infections to invasive diseases such as septicemia, necrotizing soft-tissue infections (NSTI), and streptococcal toxic shock syndrome (1). The innate immune system is the first response to *S. pyogenes* infections. To counteract this and maintain colonization, *S. pyogenes* is equipped with an arsenal of virulence factors (for an excellent review, see Ref (2)). One of these is protein SIC (Streptococcal Inhibitor of Complement). SIC is a highly polymorphic 31 kDa negatively charged protein that is secreted by only a few M serotypes including M1, M12, M55, and M57. Originally, SIC was described to interfere with complement function (3, 4), but the last decade has demonstrated that it exerts multifunctional activities and binds to several different ligands (5–9). Perhaps most importantly SIC blocks the cytolytic activity of several antimicrobial peptides (AMPs) such as LL-37, defensins, H-kininogens, H-kininogen-derived peptides, and lysozyme (5–9). The binding of SIC to cationic AMPs is mediated by the negative charge of the streptococcal protein. It has been reported that upon binding to SIC, AMPs lose their cytolytic activity (6, 7). This is considered as an important mechanism for host colonization as it prevents bacterial degradation. Besides interfering with AMPs and complement function, SIC also blocks contact activation, prolongs blood clotting, and inhibits the release of bradykinin, a potent vasodilator (10). The *sic* gene is regulated by the multiple gene regulator of GAS (mga) (11). Mga is a DNA-binding protein that activates genes important for invasion. Apart from SIC, mga also induces transcription of genes coding for proteins involved in adherence, internalization, and immune evasion (11).

Previous *in vitro* studies have shown that SIC can be considered as an anti-inflammatory and anti-cytolytic agent (3, 4, 6–10, 12). In addition, *in vivo* experiments in mice have demonstrated that a SIC-mutant strain is less virulent than a wild-type strain expressing SIC (7). During deep tissue infections such as NSTI, neutrophils are recruited to the site of infection where they degranulate and release their nuclear content also referred to as neutrophil extracellular traps (13). Neutrophil extracellular traps consist of DNA and proteins of which the majority are histones (14). It was recently reported that the released histones contribute to bacterial killing and exerts immunomodulatory functions such as the induction of cytokine/chemokine production (15–18). Thus, extracellular histones act as danger signals, alerting the innate immunity of the intruder by interacting with toll-like receptors (TLRs). Besides cytokine/chemokine production, histones can cause platelet aggregation through forming complexes with fibrinogen, thrombin generation, NLRP3 inflammasome activation, and trigger lysis of host cells and erythrocytes (17–21). How histones exert their antimicrobial activity is not completely understood. However, Hirsch (22) described how histones adsorbed microbes in a pH-dependent manner. Histones are cationic in their amino-terminal portion and neutrally charged in their carboxy termini. While the cationicity in histone H1 is predominantly constituted by lysine, the cationic charge of histone H2A, H2B, H3, and H4 is mediated by lysines and arginines. It is therefore tempting to speculate that histones could interact with SIC in a charge-dependent fashion. However, additional studies are required to unravel this issue.

Natural infections with *S. pyogenes* only occur in humans, and in the present work we investigate the immunomodulatory properties of SIC in human blood. Here, we demonstrate that SIC has multiple effects on the host response. First, this paper describes the biochemical properties of SIC/histone interactions and then continues with its functional effects on the innate immune system. We find that besides inhibiting the bactericidal activity of extracellular histones, SIC forms complexes with histones *in vitro* and colocalizes in patients with NSTI. Furthermore, our data show that SIC/histone complexes induce a broad pathological pro-inflammatory cytokine response in human blood.

## Materials and Methods

### Patient Material

Tissue biopsies were collected from patients with NSTI enrolled at Rigshospitalet, Copenhagen, Denmark, as part of the EU-funded project INFECT during 2013–2014 (ClinicalTrials.gov Identifier: NCT01790698). Also included were biopsies of healthy skin tissue obtained at plastic surgery at Karolinska University Hospital, Stockholm, Sweden. The studies were conducted in accordance with the Helsinki Declaration and approved by the regional Ethical Review Board at the Regional Ethics committee in Stockholm as well as by the National Committee on Health Research Ethics in Copenhagen. Written informed consent was obtained from all patients or their legal surrogates.

### Proteins and Antibodies

Histone H1, H2A, H2B, H3.1, and H4 were purchased from Bionordika (Stockholm, Sweden) and calf thymus histones (histones) from Roche (Basel, Switzerland). [Histone H4-derived peptides were synthesized at Biopeptide Co. (San Diego, CA, USA).] Peroxidase-conjugated goat anti-rabbit and goat anti-mice immunoglobulin G were from Bio-Rad Laboratories (Berkley, CA, USA). Antibodies against histone H4 (polyclonal chip-grade anti-histone H4) were from Abcam (Cambridge, UK). Rat polyclonal antibodies to human TLR4, TLR2 and isotype control and the TLR4-inhibitor CLI-095 were purchased from InvivoGen (San Diego, CA, USA).

### Bacterial Strains and Protein Purification

The *S. pyogenes* strain AP1 (M1 serotype) was from World Health Organization Collaborating Center for Reference and Research on Streptococci, Prague, Czech Republic. SIC was purified from *S. pyogenes* AP1 strain culture medium as described by Åkesson et al. (3) by precipitation of the bacterial supernatant (SN) with 30% ammonium sulfate, followed by ion-exchange chromatography on a Mono Q column. SIC fractions were dialyzed against 20-mM Tris–HCl pH 7.5 and analyzed using SDS-PAGE. The SIC-mutant strain was generated as described by Frick et al. (7). The SIC preparation used in this study was analyzed by shotgun mass spectrometry. No other streptococcal proteins were detected and the only contamination found were traces of human keratin. Antiserum against SIC and isotype control serum were raised in rabbits. SIC was labeled with ^125^I using Pierce IODO.BEADS^®^ Iodination reagent (Thermo Scientific, Waltham, MA, USA). Molarity calculation of SIC was based on a molecular weight of 31,000 Da.

### Blood Collection

Blood was drawn from healthy donors into 6.0-mL sodium heparin tubes (Becton Dickinson, Franklin Lakes, NJ, USA) or into 2.7-mL 0.109-M sodium citrate tubes (Becton Dickinson, Franklin Lakes, NJ, USA). Blood samples were stored at room temperature for 30 min before usage.

### SIC Precipitation, SDS-PAGE, and Western Blotting

*Streptococcus pyogenes* AP1 bacteria and SIC-mutant bacteria were grown for 9 h in Todd–Hewitt (TH) broth at 37°C. After 6 h, the bacteria were centrifuged at 2,000× *g* and the SNs were sterile filtered. SNs (1 mL) from SIC-producing AP1 and the SIC knockout were mixed with 250 µL of 100% trichloroacetic acid. Samples were incubated for 10 min at 4°C. All samples were centrifuged at 18 000× *g* for 5 min. The SN was removed from the white pellet in bottom of every test tube. Pellets were dissolved in 200 µL ice-cold 100% acetone. All samples were centrifuged again at 14,000 rpm for 5 min. SN was removed and the pellet was dried at 95°C in a heat block for 10 min. Each pellet was dissolved in sample buffer (Bio-Rad Laboratories, Berkeley, CA, USA) and boiled for 10 min at 95°C. Samples were loaded into a 10% PAGE as described by Laemmli (23). Proteins were transferred onto a polyvinylidene difluoride membrane (GE Healthcare, Uppsala, Sweden), blocked with 5% non-fat milk powder in 10-mM PBS, 0.05% Tween-20 (PBS-T), incubated with primary rabbit anti-SIC serum (1:1,000), and secondary peroxidase-labeled goat anti-mouse IgG (1:3,000, Bio-Rad Laboratories, Berkley, CA, USA). All incubation steps were performed at RT for 60 min on rotation and were followed by three washing steps in PBS-T. The polyvinylidene difluoride membrane was developed using SuperSignal West Pico Chemiluminescence kit (Thermo Scientific, Waltham, MA, USA) according to manufacturer’s instructions.

### Slot Blot

A polyvinylidene difluoride filter (Millipore, Billerica, MA, USA) was activated in methanol, washed in destilled water, and exposed to different histones subtypes in three different concentrations using a Milliblot-D system (Millipore, Billerica, MA, USA). The filter was washed with 100-µL PBS, blocked with 5% non-fat milk powder in PBS and probed with ^125^I-labeled SIC (2 × 10^5^ cpm/mL) for 3 h in RT. The filter was washed three times for 20 min with PBS-T and autoradiography was carried out using Kodak x-Omat AR films and regular intensifying screens.

### Peptide ELISA

Microtiter plates were coated overnight with 100 µL histone H4-derived peptides (1 µM) in coating buffer (15.9-mM Na_2_CO_3_, 30-mM NaHCO_3_, pH 9.6) at 4°C. The next day, plates were washed three times in PBS containing 0.05% Tween-20 (PBST) and blocked in PBST containing 0.5% bovine serum albumin for 60 min at 37°C. After blocking and another washing step, plates were probed with 100 µL SIC (40 nM) for 1 h at 37°C and binding was detected with 100 µL rabbit SIC antisera (1:2,000) followed by 100 µL of a peroxidase-conjugated antibody against rabbit IgG (1:3,000, Bio-Rad Laboratories, Berkeley, CA, USA). Stabilized tetramethylbenzidine Chromogen (Thermo Fischer Scientific, Waltham, MA, USA) was used as a substrate for the peroxidase-conjugated antibody. The reaction was terminated with 10% H_2_SO_4_ and measured at 450 nm for 0.1 s per measured well.

### Surface Plasmon Resonance

All analyses were performed using a BIAcore X100 instrument (GE Healthcare, Uppsala, Sweden). Histone H4 was diluted in sodium acetate (10 mM, pH 5.5) and immobilized to a Sensor Chip CM5 *via* amine coupling to flow cell 2. Flow cell 1 was immobilized without protein and was used as a control for each experiment. SIC (400–25 nM) was injected in a flow (30 µL/min) over the Sensor Chip in HBS-EP buffer [10-mM HEPES, 150-mM NaCl, 3-mM EDTA, 0.05% (v/v) Surfactant P20, pH 7.4]. Regeneration of the Sensor Chip was obtained by injection of 30 µL 50-mM NaOH. All experiments were run at 25°C in degassed buffers. Affinity constants were calculated using BIAevaluation software (GE Healthcare, Uppsala, Sweden).

### Negative Staining and Transmission Electron Microscopy

Binding between SIC and histone H4 was visualized using negative staining and electron microscopy as previously described (24). Histone H4 was conjugated with 10-nm colloidal gold particles according to routine protocols (25). Conjugates were incubated with each other for 30 min at RT and negatively stained with 0.75% uranyl formate. Specimens were examined in a Philips/FEI CM100 BioTwin transmission electron microscope at a 100,000× magnification.

### Bacterial Viable Count Assay

*Streptococcus pyogenes* AP1 strain and the SIC-mutant *S. pyogenes* strain were plated overnight at 37°C on blood agar plates and stored at 4°C. For each experiment, colonies from the blood agar plate were added to 10 mL in TH broth (Sigma-Aldrich, St. Louis, MO, USA) and incubated at 37°C overnight. SIC mutant was grown in TH broth containing 0.15-mg/mL Kanamycin. The following day, the bacteria were mixed and 200 µL were transferred to 10-mL fresh TH broth and incubated to reach logarithmic phase (OD_620nm_ 0.4) at 37°C. The bacteria were centrifuged at 2,000× *g* and washed in ice cold 10-mM PBS with 5-mM glucose (PBS-G). Bacteria were diluted to 2 × 10^6^ colony-forming units (CFUs)/mL in PBS-G. Histones (5 µg/mL) or histone H4 (0.36 µM), with or without increasing concentrations of SIC (0.16, 0.32, or 0.64 µM), were preincubated for 30 min at 37°C in a total volume of 50 µL. The same volume of diluted bacteria was added to each sample, and the samples were incubated for 60 min at 37°C with 5% CO_2_. All samples were diluted in PBS-G and plated on TH agar plates overnight at 37°C. The following day, colonies were manually counted and the number of viable CFUs was described as a ratio of viable colonies compared with non-stimulated bacteria.

### Hemolysis Assay

Citrated blood (1 mL) was centrifuged at 2,000× *g* for 10 min to pellet cells. The blood plasma was discarded and replaced by an equal volume of PBS. This washing procedure was repeated twice. Histones and SIC were preincubated for 30 min at 37°C and erythrocytes were then added to each sample in a final dilution of 5% (v/v). After 60 min incubation at 37°C on rotation, all samples were centrifuged at 5,000× *g* for 10 min. The SN was transferred to a 96-well plate (Thermo Scientific, Waltham, MA, USA) and hemolysis was measured at 540 nm. Histone-induced hemolysis was presented as percentage of lysis buffer (0.1% Triton X-100)-induced hemolysis.

### Cytokine Production in Blood

Histones (50 µg/mL), LL-37 (25 µg/mL), β defensin-3 (25 µg/mL), high-mobility group box 1 (HMGB1, 25 µg/mL), heparin-binding protein (HBP, 25 µg/mL), SIC (0.64 µM), or the AMPs in combination with different SIC concentrations were incubated with heparinized blood diluted 1:1 in PBS for 7 h at 37°C on rotation. For inhibition experiments, antibodies against human TLR4 (25 µg/mL), human TLR2 (25 µg/mL), isotype control (25 µg/mL), or the TLR4 inhibitor CLI-095 (1–10 µg/mL) were added simultaneously to histones and SIC. Blood aliquots were removed at different time points and centrifuged at 2,000× *g* for 10 min. The plasma SN was removed and frozen at −20°C. IL-6 was measured in heparin plasma SN using Human IL-6 Quantikine ELISA kit (R&D Systems, Minneapolis, MN, USA) using the manufacturer’s instructions. Samples were discarded after the IL-6 measurements and were not repeatedly frozen.

### Immunostaining of Tissue Biopsies

Snap-frozen tissue biopsies from healthy controls or from patients with NSTI caused by M1 or M3 *S. pyogenes* strains were analyzed. The biopsies were cryosectioned to 8 µm and fixed in ice-cold acetone or 2% formaldehyde for immunofluorescent or immunohistochemical stainings, respectively. Stainings were performed in singlets on consecutive sections as described previously (16, 17, 26, 27). The following antibodies were used at predetermined optimal concentrations: mouse monoclonal anti-histone H4 (Abcam, Cambridge, UK), rabbit anti-SIC serum, goat polyclonal anti-Lancefield group A carbohydrate (Abcam, Cambridge, UK), and rabbit polyclonal antibodies against human HMGB1 (Abcam, Cambridge, UK) and human IL-8/NAP-1 (Invitrogen, Waltham, MA, USA). Biotinylated secondary antibodies included rabbit-anti-goat IgG and goat-anti-rabbit IgG (both from Vector Laboratories, Burlingame, CA, USA). For fluorescence stainings, Alexa 546 conjugated donkey anti-rabbit IgG and Alexa 488 conjugated donkey anti-mouse IgG (both from Molecular Probes, Eugene, OR, USA) were used. Slides were mounted using DAPI-supplemented mounting media (Molecular Probes, Eugene, OR, USA). Single stainings were performed to assure specificity of staining patterns. For image evaluation, a Nikon A1R confocal microscope was used (Nikon Instruments, Amstelveen, the Netherlands). The immunohistochemically stained sections were analyzed by acquired computerized image analysis (ACIA) as previously described (28). The cell area was defined by the hematoxylin counterstaining and the results are presented as ACIA values, which equal the percentage of positively stained area × mean intensity of positive staining.

### Multiplex Cytokine Quantification Assay

The cytokine analysis was performed using a Bio-Plex 200 instrument and a Bio-Plex Pro Human Cytokine 27-Plex Assay (both from Bio-Rad Laboratories, Berkeley, CA, USA) FGF basic, Eotaxin, G-CSF, GM-CSF, IFN-γ, IL-1β, IL-1ra, IL-2, IL-4, IL-5, IL-6, IL-7, IL-8, IL-9, IL-10, IL-12, IL-13, IL-15, IL-17, CXCL10, CCL2, CCL3, CCL4, PDGF-bb, RANTES, TNF-α, and VEGF. The cytokine panel was designed to provide a measure of cytokines, chemokines, and growth factors. All samples, standards, and controls were run in duplicates and data was acquired using Bio-Plex Manager Software (version 6.1).

### Statistical Analyses

Data were analyzed using GraphPad Prism 7 (GraphPad Software, San Diego, CA, USA). One-way ANOVA with Dunnett’s or Tukey’s test was used to determine significance between multiple comparisons.

## Results

### Expression of SIC and Binding of SIC to Extracellular Histones

**Figure 1:**
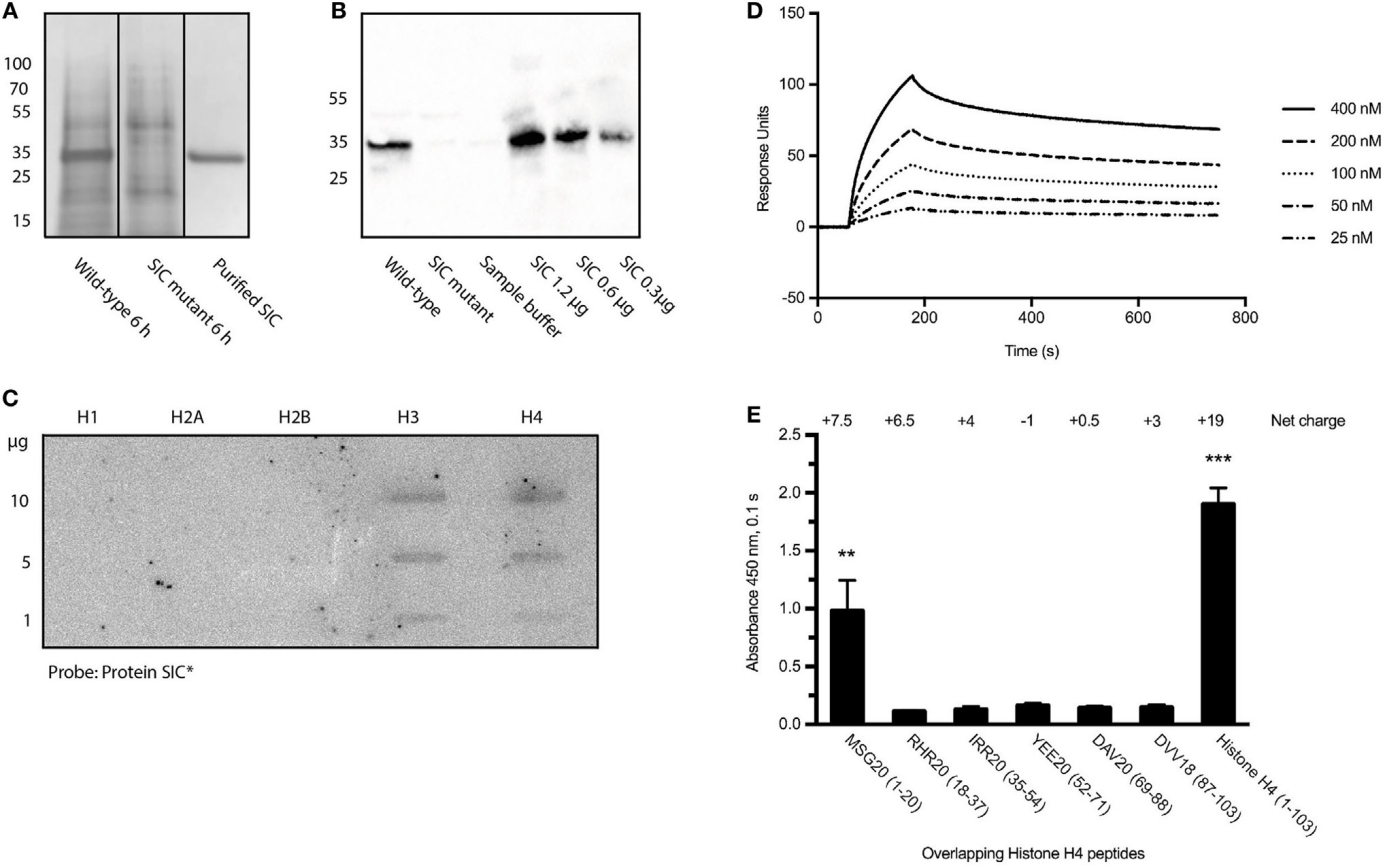
Streptococcal inhibitor of complement (SIC) expression and its binding to histones. **(A)** *S. pyogenes* wild-type and SIC-mutant SNs were precipitated with trichloroacetic acid and separated on a 10% SDS-PAGE. **(B)** SIC was identified in SNs using rabbit antisera against SIC. **(C)** An Immobilon filter was immobilized with different histone subtypes, incubated with ^125^I-labeled SIC and bound probe was detected with the Fuji FLA-3000 imaging system. **(D)** SPR technology was used to show the interaction between histone H4 and SIC. Histone H4 was coated on a CM5 Sensor Chip and SIC was applied in a flow at different concentrations. **(E)** Microtiter plates were coated with histone H4-derived peptides and probed with SIC. Binding was detected using rabbit antisera against SIC and visualized by a peroxidase-conjugated secondary antibody. Data show means ± SEM (one-way ANOVA, Tukey’s test). All experiments were independently performed at least three times and one representative image is shown. *P ≤ 0.05, **P ≤ 0.01, and ***P ≤ 0.001.

Before investigating the interaction between SIC and histones, the production of the streptococcal protein in the strains employed in this study was determined. The *S. pyogenes* isolates AP1 (wild-type) and the isogenic *S. pyogenes*-mutant strain-lacking SIC (SIC-mutant) were used throughout this study. The SNs from both bacterial strains were collected at 6 h, precipitated with trichloroacetic acid, and SIC was visualized using SDS-PAGE (Figure 1A; Figure S1A in Supplementary Material) followed by Western blot analysis using antisera against SIC (Figure 1B; Figure S1B in Supplementary Material). The results show that SIC is secreted from the wild-type, but not expressed in the SIC-mutant strain, and they also reveal that SIC is the most abundant protein secreted from *S. pyogenes* under these culture conditions (Figure 1A). No SIC expression was detected when the mutant strain was analyzed (Figures 1A,B).

**Figure 2:**
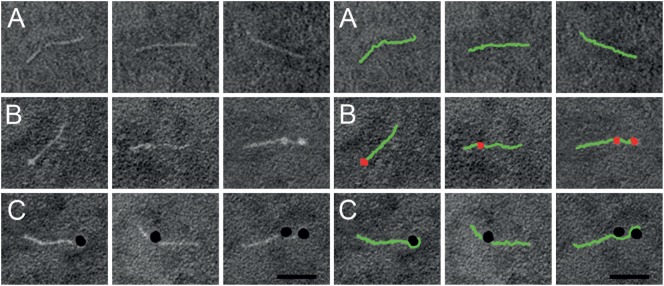
Negative staining electron microscopy of SIC in complex with histone H4. Right panel shows a duplicate of the left panel, but with SIC (green) and histone H4 (red) illustrated in pseudocolors. **(A)** Linear SIC monomers in the absence of a ligand. **(B)** Histone H4 was added to SIC and prepared for negative staining electron microscopy. SIC monomers are depicted with one or two bound molecules of histone H4. **(C)** Histone H4 was labeled with 10-nm colloidal gold and added to SIC. SIC monomers binds to one or two molecules of histone H4 conjugated to gold particles. Scale bar 25 nm.

Previous work has shown that SIC is highly anionic and interacts with a number of different AMPs (5–7). As extracellular histones are, like AMPs, positively charged, we sought to study a possible interaction between SIC and histones. The five different histone subtypes were immobilized in different concentrations on an Immobilon filter and probed with ^125^I-labeled SIC. The slot blot depicted that SIC interacts with histones H3 and H4 but not with histones H1, H2A, or H2B (Figure 1C). Histone H4 is highly cationic (net charge + 19) and it was recently described to trigger a number of pathological reactions such as the aggregation of platelets, hemolysis of erythrocytes, and the production of chemokines under *in vitro* and *in vivo* conditions (15–19, 29). Based on these results, we decided to focus on histone H4 in subsequent experiments. To this end, we first confirmed the interaction between SIC and histone H4 using Surface Plasmon Resonance technology. These experiments revealed that SIC and histone H4 associate rapidly followed by a slow dissociation phase, resulting in an affinity constant which was in the lower nanomolar range (Table 1 and Figure 1D). To map the SIC/histone H4 interaction site, overlapping histone H4-derived peptides were coated on a 96-well plate, incubated with SIC and immunodetected with antisera against SIC. The cationic charge distribution of histone H4 is localized to its *N*-terminus. This site, covered by peptide MSG20 (amino acids 1–20), is the only peptide that had affinity for SIC (Figure 1E). The binding between SIC and histone H4 was also analyzed by negative staining electron microscopy. SIC has three structural domains; a short repeat region, a long repeat region, and a proline-rich region (3, 12). These three domains form a linear structured protein (Figure 2A) that provides two distinct binding sites for histone H4. In complex with SIC, histone H4 appears as a globular folded protein (Figure 2B). Notably, binding of histone H4 did not change the conformation of SIC. In the next series of experiments, histone H4 was labeled with colloidal gold and its interaction with SIC was visualized (Figure 2C). While monomeric negatively stained SIC is depicted in Figures 2A–C, the vast majority of SIC and histone H4 were found in clusters suggesting that they may form larger aggregates (Figure S2A in Supplementary Material) or oligomers (Figure S2B in Supplementary Material). Taken together, the results underline that SIC is a highly abundant secreted protein in the *S. pyogenes* M1 strains, and that the protein specifically interacts with histones H3 and H4, but not with histones H1, H2A, and H2B, respectively. Moreover, we found that SIC binds to the N-terminal part of histone H4, which is the most cationic part of the protein, and can form complexes with up to two histone molecules simultaneously.

**Table 1:** Affinity constants for the binding of SIC to histone H4.

Sample	*k_a_*(1/Ms)	*k_d_*(1/s)	*K_D_*(M)
Protein SIC	3.347 × 10^4^	5.908 × 10^−4^	1.765 × 10^−8^

*Histone H4 was immobilized to a Biacore CM5 Sensor Chip and different concentrations of SIC were applied in a flow. Affinity constants for SIC/histone H4 binding were determined using BIAevaluation software (GE Healthcare, Uppsala, Sweden)*.

### SIC Interfering with Antimicrobial and Hemolytic Activity of Extracellular Histones

**Figure 3:**
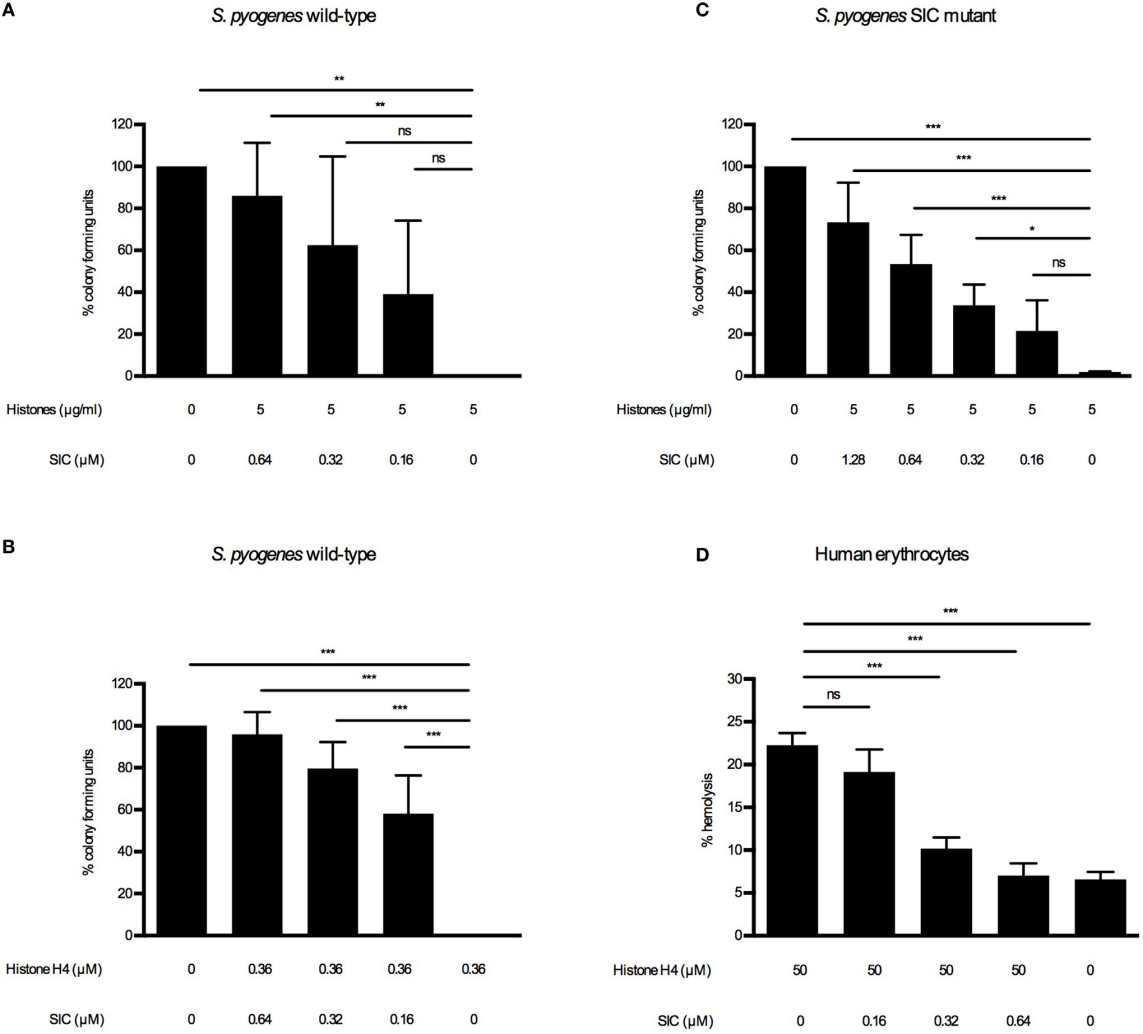
SIC inhibiting the cytolytic activity of histones. *S. pyogenes* wild-type were grown to mid-log phase, washed, and diluted to 2 × 10^6^ CFU/mL. Histones **(A)** and SIC or histone H4 **(B)** and SIC were preincubated for 1 h, and mixed with the wild-type bacteria for 1 h at 37°C. **(C)** *S. pyogenes* SIC mutant was prepared as described above. Histones and SIC were preincubated for 1 h, and mixed with the SIC mutant for 1 h at 37°C. Samples were diluted and plated on TH agar plates and CFU were determined the following day. Data show mean ± SEM (one-way ANOVA Dunnett’s test). **(D)** Erythrocytes were diluted to 5% (v/v) in PBS and incubated with histones and different concentrations of SIC for 1 h. Hemolysis was measured at 540 nm in supernatants after centrifugation, and was expressed as percentage of erythrocytes treated with 0.1% Triton X-100. Data show mean ± SEM (one-way ANOVA Dunnet’s test). All experiments were independently performed at least three times. *P ≤ 0.05, **P ≤ 0.01, and ***P ≤ 0.001.

Extracellular histones and other AMPs are potent killers of a broad range of bacterial pathogens (22, 30, 31). To counteract a cytotoxic attack by such substances, *S. pyogenes* has developed several strategies to cope with these host responses, including the secretion of SIC. It was previously reported that SIC inhibits the bactericidal activity of LL-37 (7) and several defensins (5, 7) and it also interferes with the contact and complement systems (6, 10). In the case of AMPs, it has been reported that SIC prevents their interaction with the bacterial membrane (3–10). Based on these findings, we sought to determine whether SIC interferes with the bactericidal activity of histones. To investigate the effect of SIC on histones, we performed viable count assays. Wild-type bacteria were grown to log-phase, extensively washed, and diluted to enable determination of viability by measuring CFUs. As the washing steps let to a depletion of endogenously produced SIC, resuspended bacteria were reconstituted with SIC purified from the wild-type SN. Reconstituted and non-reconstituted wild-type *S. pyogenes* bacteria were incubated with histones or histone H4. After 60-min incubation, bacteria were plated and enumerated the next day to determine the viability. Addition of SIC rescued bacteria in a dose-dependent manner against histones (Figure 3A) and histone H4 (Figure 3B) and similar results were obtained when the SIC mutant was analyzed (Figure 3C). Besides perforating bacterial membranes, extracellular histones can lyse eukaryotic cells including erythrocytes and thereby cause hemolysis, a common characteristic of systemic inflammatory response syndromes (17). To investigate if SIC can block histone-induced hemolysis, erythrocytes were washed and diluted in PBS. Histones were then added to induce hemolysis. In the absence of SIC, histone-induced hemolysis occurred after 60 min while increasing concentrations of SIC rescued erythrocytes from histone-induced hemolysis (Figure 3D). These results suggest that SIC neutralizes the lytic activity of extracellular histones toward both bacterial and human cell membranes.

### SIC/Histone Complexes Triggering the Induction of Multiple Cytokines and Chemokines

**Figure 4:**
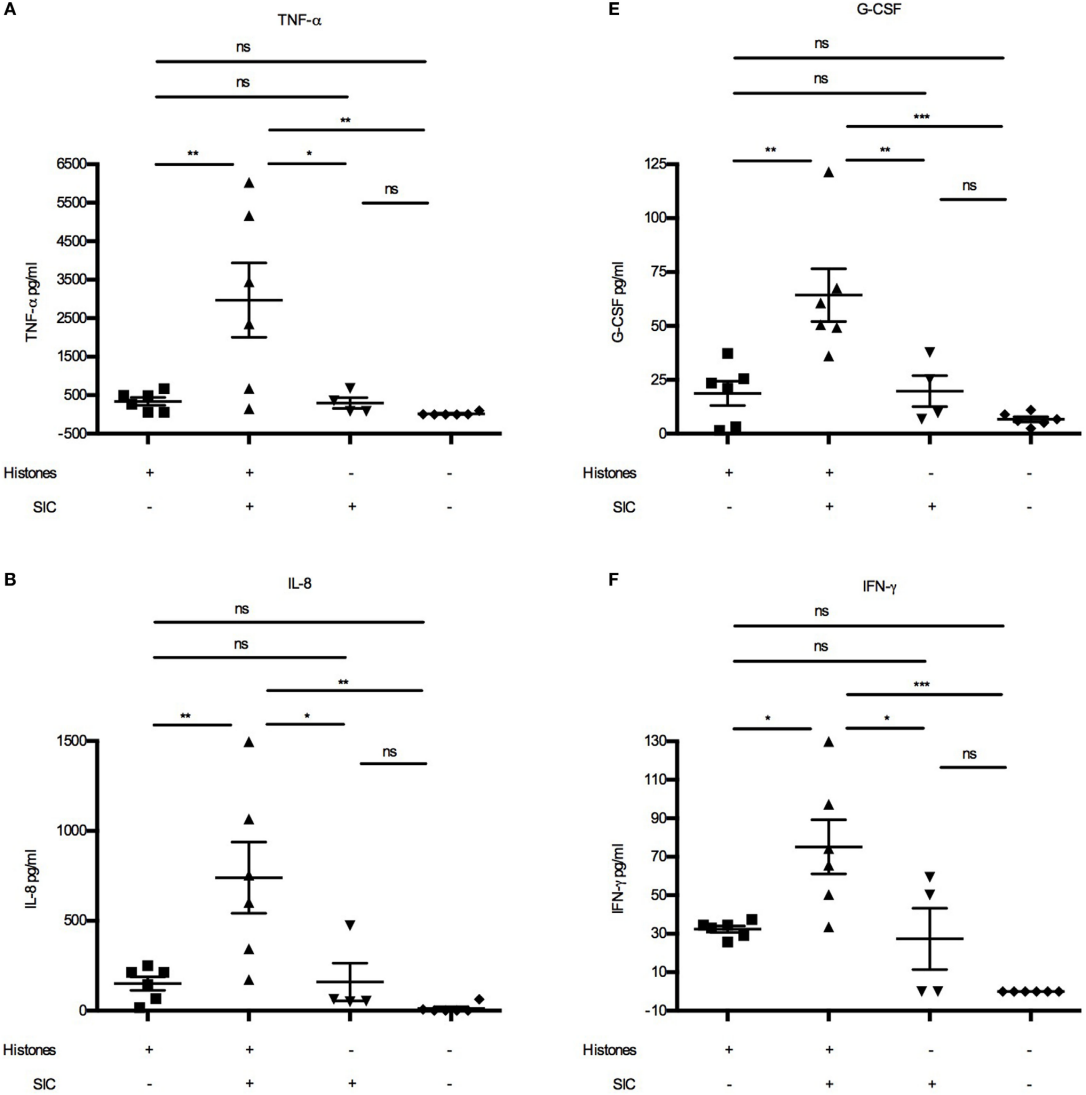

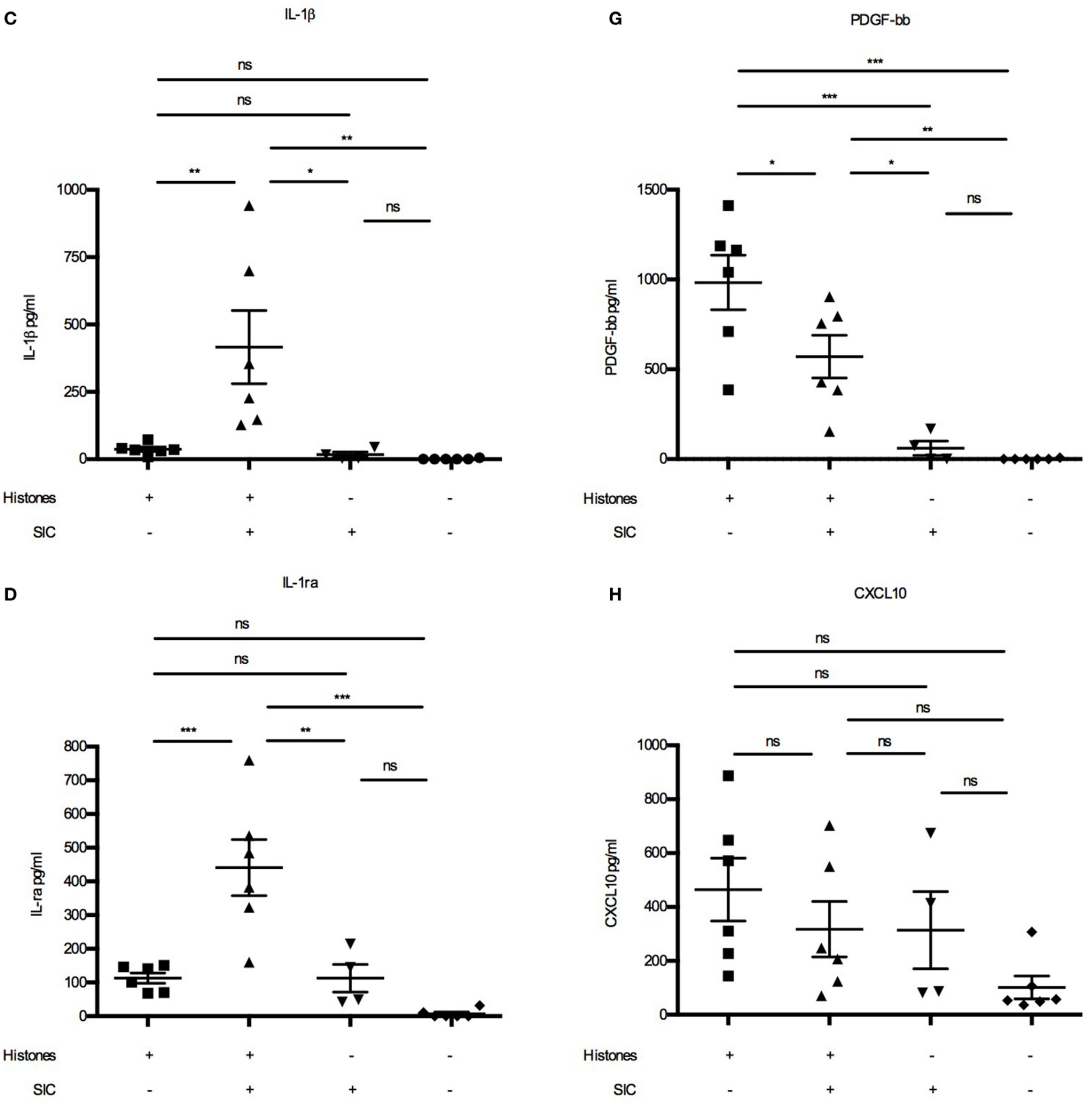
SIC/histone complexes inducing the release of a broad range of cytokines and chemokines. Heparinized blood diluted 1:1 in PBS was stimulated with either histones (*n* = 6), histones with SIC (*n* = 6), SIC alone (*n* = 4), or PBS (*n* = 6) for 7 h at 37°C. Plasma SNs were analyzed for a panel of cytokines, chemokines, and growth factors including TNF-α **(A)**, IL-8 **(B)**, IL-1β **(C)**, IL-1ra **(D)**, G-CSF **(E)**, IFN-γ **(F)**, PDGF-bb **(G)**, and CXCL10 **(H)** using a Bio-Plex cytokine quantification assay. Data show mean ± SEM (one-way ANOVA, Tukey’s test). All samples were measured in duplicates. *P ≤ 0.05, **P ≤ 0.01, and ***P ≤ 0.001.

**Figure 5:**
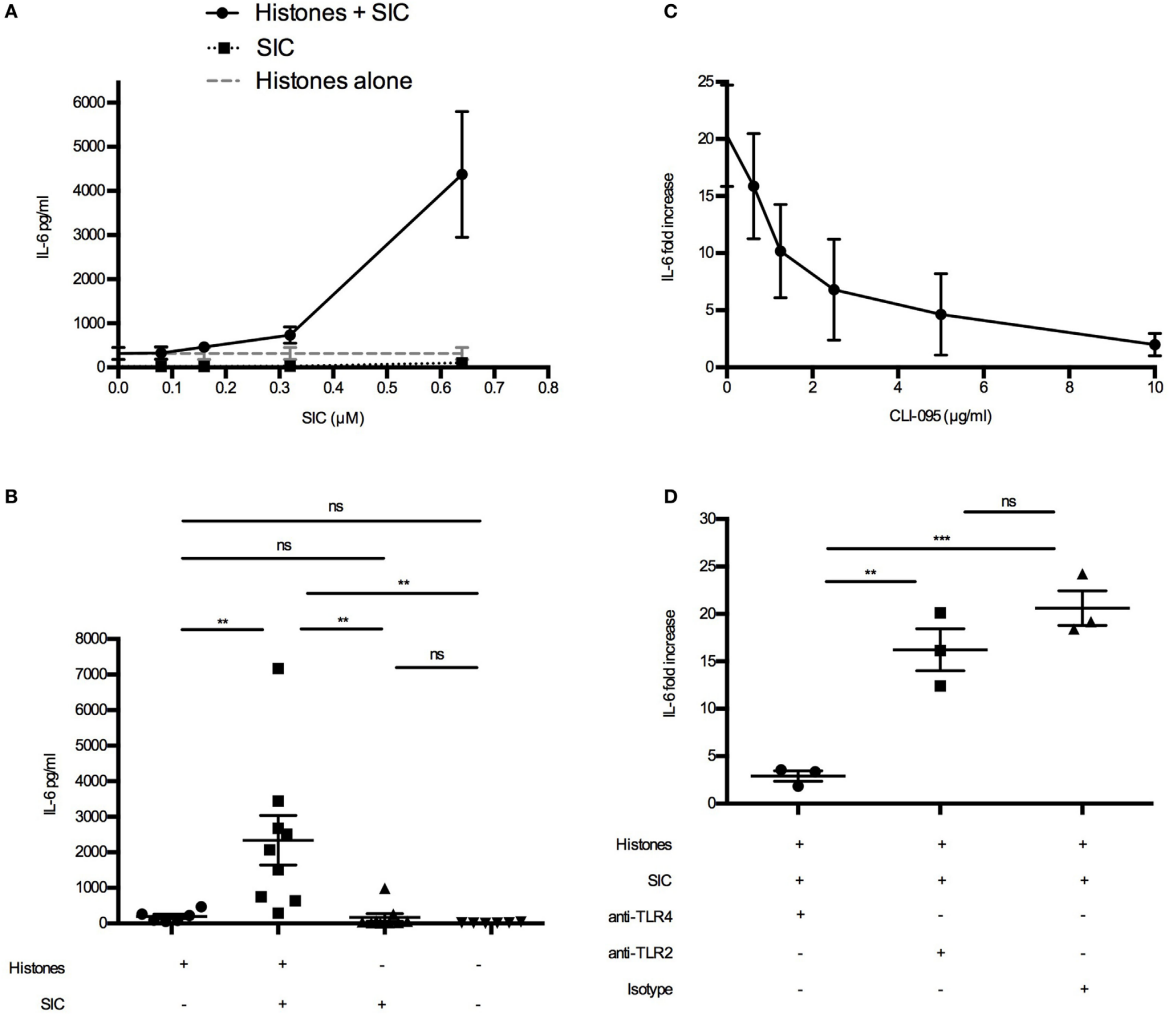
SIC/histone complexes inducing IL-6 production *via* TLR4. **(A)** Heparinized blood was diluted 1:1 in PBS. Histones (50 µg/mL) and different concentrations of SIC were preincubated for 1 h and added to the blood. Samples were incubated for 7 h at 37°C and IL-6 was measured in the plasma SN. Data show mean ± SEM (*n* = 3). **(B)** Heparinized blood from six donors was investigated for IL-6 production after incubation with SIC/histone complexes for 7 h at 37°C. Data show mean ± SEM (one-way ANOVA, Tukey’s test). IL-6 production was measured in blood incubated with histones, SIC, and different concentrations of the TLR4 inhibitor CLI-095 (*n* = 3) **(C)** or anti-TLR4 antibodies (25 µg/mL) (*n* = 3), anti-TLR2 antibodies (25 µg/mL) (*n* = 3), or isotype control (25 µg/mL) (*n* = 3) **(D)**. Values are expressed as fold increase to diluted blood stimulated with PBS. Data show mean ± SEM (one-way ANOVA, Tukey’s test). *P ≤ 0.05, **P ≤ 0.01, and ***P ≤ 0.001.

Extracellular histones were recently described to act as danger-associated molecular patterns (or danger signals) as they can induce cytokine (15) and chemokine production (16) and trigger leukocyte migration *in vivo* (16). Cytokines such as TNF-α, IL-6, IL-8, CXCL10, and IFN-γ are released upon histone stimulation, and it has been reported that their release is dependent on different TLRs (15, 16, 20, 21). As our data demonstrated that SIC can block the cytolytic activity of histones, we wished to investigate whether it also inhibits histone-induced release of these inflammatory mediators. Using Bioplex multiplex assays, blood from healthy donors were incubated with histones in the absence or presence of SIC and a panel of 27 cytokines, chemokines, and growth factors were measured. Under these experimental conditions, histones alone acted often as weak or moderate inducers of cytokines/chemokines (Figure 4). However, histones in complex with SIC boosted the pro-inflammatory response, yielding a robust increase of cytokines/chemokines such as TNF-α (Figure 4A), IL-8 (Figure 4B), IL-1β (Figure 4C), IL-1ra (Figure 4D), G-CSF (Figure 4E), IFN-γ (Figure 4F), and IL-6 (Figures 5A,B). SIC alone had no effect on the induction of these proteins except for one donor. Interestingly, the coincubation with SIC did not always boost the inflammatory response. For example, histone-induced PDGF-bb expression was to some extent blocked by SIC (Figure 4G) and SIC did not affect histone-induced CXCL10 production (Figure 4H). Furthermore, a broad range of cytokines were not affected by either histones or SIC, or not significantly elevated (Figure S3 in Supplementary Material). This shows that SIC together with histones induced a potential pathological response of a broad range of cytokines and chemokines.

### SIC Increasing Histone-Induced IL-6 Production *via* TLR4

To investigate the target receptor of SIC/histone-induced cytokine production, we focused on IL-6. First we confirmed, using blood from one donor, that SIC/histone-complexes can induce IL-6 production (Figure 5A). Interestingly, the IL-6 production was amplified when the SIC concentration was increased (Figure 5A). Then we repeated the experiment using several donors at the most potent SIC concentration (Figure 5B). Our previous studies have shown that histones induce chemokine production by binding to the TLR4/MD-2 complex (16). To investigate whether this pathway is responsible for the enhancing effect, SIC and histones were added to human blood together with the TLR4-inhibitor CLI-095 or inhibitory antibodies against TLR4, TLR2, or an isotype control. Figures 5C,D depict that blocking the TLR4 response almost completely abrogated IL-6 production, suggesting that the interaction of SIC with extracellular histones can contribute to the release of pathological levels of IL-6 involving an activation of the TLR4 signaling pathway.

To investigate whether the SIC-induced amplification of inflammation applies not only to histones, but also to other cationic SIC-binding AMPs, we incubated blood from healthy donors together with LL-37, β defensin-3, HMGB1, and HBP in the absence or presence of SIC. Subsequent Bioplex multiplex assays revealed that SIC boosted cytokine/chemokine production of LL-37, β defensin-3, and HMGB1, but had no effect on HBP (Figure S4 in Supplementary Material). These findings indicate that the pro-inflammatory property of SIC applies to several cationic AMPs, including histones, with important functions in the innate immune system.

### SIC and Extracellular Histones Present at the Infected Tissue Site in Patients with Necrotizing Soft-Tissue Infection

**Figure 6:**
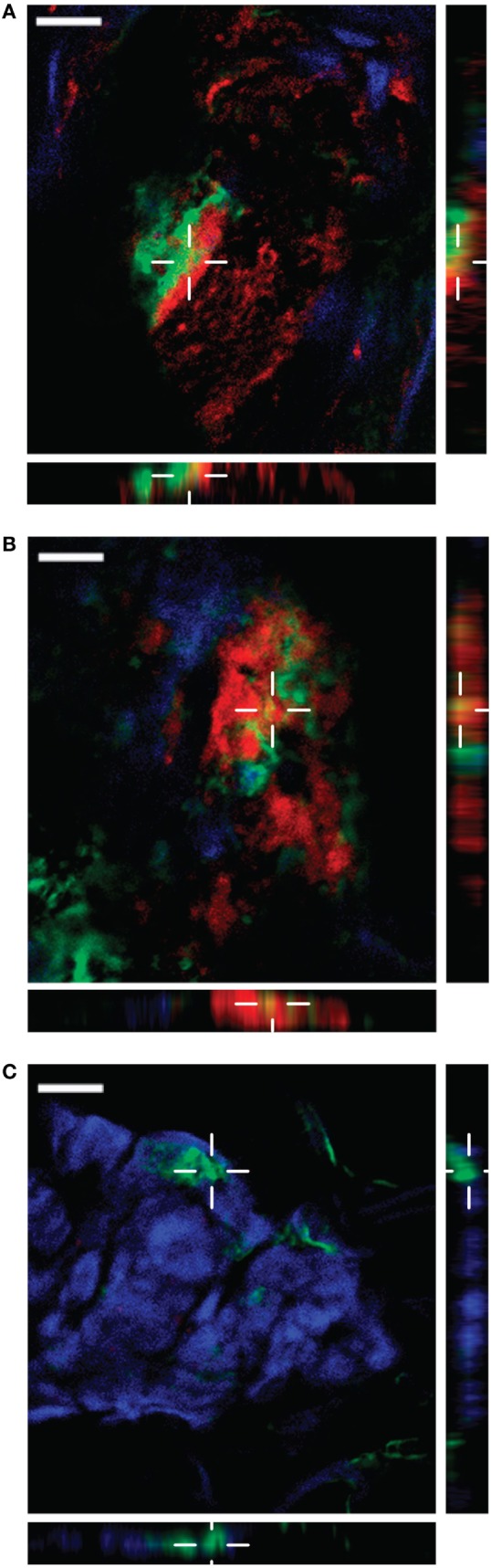
Streptococcal inhibitor of complement (SIC) expression and colocalization with histone H4 in patients with necrotizing soft-tissue infection. Snap-frozen biopsies from patients with infections caused by M1 **(A,B)** *S. pyogenes* strains were analyzed for SIC (red) and histone H4 (green) expression. DNA is stained with DAPI (blue). A biopsy from a healthy donor was used as a control **(C)**. To confirm colocalization of the two markers, vertical planes of the indicated areas are shown, demonstrating colocalization in **(A,B)**. Scale bar 20 µm.

*S. pyogenes* strains of the M1 serotype are among the most frequently identified serotypes isolated from patients with severe invasive infections including NSTI and toxic shock syndrome (32). We previously reported that in patients with NSTI, histones from necrotic cells are released into the extracellular compartments (16, 17). As invasive *S. pyogenes* strains often belong to the M1 serotype and secrete large quantities of SIC under *in vitro* conditions (7), we investigated whether SIC is released in NSTI patients, and if so, whether it is found to colocalize with histones. To this end, tissue sections from biopsies of patients infected with *S. pyogenes* M1 or M3 (a serotype negative for SIC) strains were analyzed with immunofluorescence staining. As shown in Table 2, we analyzed three biopsies that were positively stained for HMGB1, a marker for disease severity (33). All analyzed sections exhibited signs of inflammation (IL-8) and were colonized with *S. pyogenes* (GAS). Moreover, a biopsy from a healthy donor was included with undetectable levels of HMGB1, IL-8, and *S. pyogenes*. The four biopsies were stained with antibodies against SIC (red) and histone H4 (green) as well as with the nuclear DNA stain DAPI (blue). Histone H4 was readily detected in tissue sections from both M1- and M3-infected patients, as well as in the biopsy from a healthy control (Figure S5 in Supplementary Material). SIC showed a strong staining in the M1-infected tissue (Figures 5A,B in Supplementary Material), whereas in the *M*3 only a background staining was noted (Figure 5C in Supplementary Material). Moreover, we recorded a few areas of colocalization between SIC and histone H4 in the biopsy sections from M1 patients (Figures 6A,B), indicative of a potential SIC/histone H4 complex formation at the infected tissue site. Histone H4 but not SIC was detected in the biopsy from a healthy donor (Figure 6C; Figure 5D in Supplementary Material). Taken together, these findings demonstrate that SIC is expressed and released during *S. pyogenes* NSTI and that SIC is present in areas with extracellular histones and may form complexes at the infected tissue site of patients with NSTI. To conclude, our data show that SIC can form complexes with histones *in vitro*, and block histone-mediated effects on both bacterial and eukaryotic membranes. Furthermore, we found that SIC/histone complex formation synergistically increases a broad and potentially pathological inflammatory response in human blood. Lastly, we demonstrate that SIC is expressed in patients suffering from NSTI and that SIC partly colocalize with histones, suggesting that complex formation also occurs in patients with invasive streptococcal skin infections.

**Table 2:** Analysis of biopsies from patients with *S. pyogenes* necrotizing soft-tissue infections.

Patient ID	M type	HMGB1	IL-8	GAS
2001	M1	30	51	24
2006	M1	47	81	65
2028	M3	57	65	51
Healthy control	Negative	Negative	Negative	Negative

*Biopsies were stained with antibodies against high-mobility group (HMGB1), IL-8, and the Lancefield group-specific carbohydrate identifying group A *Streptococcu*s (GAS), and the stainings were quantified using acquired computerized image analysis*.

## Discussion

In the present study, we found that SIC can boost pro-inflammatory responses in human blood by forming complexes with histones. The streptococcal protein was initially recognized for its ability to inhibit the complement system by binding to the C5b–C9 complex, also known as the membrane attack complex, hence the name streptococcal inhibitor of complement (3). Although this interaction has been intensively studied, the pathophysiological role is not completely understood. Notably, *S. pyogenes* is resistant to complement-mediated lysis because of its thick peptidoglycan layer (4). These findings suggest that SIC may employ additional functions beyond blocking the complement system. For example, the extensive list of AMPs including extracellular histones constitutes another important group of SIC ligands. AMPs, such as LL-37 and defensins, are expressed by epithelial cells in the upper respiratory tract (34, 35). Despite this antimicrobial environment, *S. pyogenes* colonizes the pharynx of up to 30% of the population without being killed.

Within the last years, there have been a couple of studies showing that the interaction between SIC and host-derived proteins helps the bacteria to escape elimination by host defense systems. For instance, streptoccocal survival in saliva is decreased when SIC-mutant strains are employed (36) and similar findings were published using a murine model of oropharyngeal infection (37). Based on these and other reports, we hypothesize that under these or other non-inflammatory conditions, SIC is needed to protect streptococci from an otherwise lethal immune attack. Thus, it seems that SIC helps to establish a non-inflammatory environment when the bacteria need to colonize and start to proliferate. However, under invasive conditions, SIC can contribute to the induction of systemic inflammatory reactions. For instance, SIC is upregulated when exposed to blood. Maximum levels of *sic* expression is detected already 30 min after its exposure to blood (38). It should be noted that SIC levels are increased together with other mga-regulated mediators such as fibronectin-binding proteins, collagen-like surface proteins, C5a peptidase, and M-proteins. Like SIC, these proteins can evoke pro-inflammatory reaction which in concert can lead to life-threatening complications as, for instance, seen in patients suffering from streptococcal necrotizing fasciitis. During these necrotic conditions, SIC can form complexes with extracellular histones that in turn induce cytokine production, thereby causing an overwhelming and pathological inflammatory response. Using other danger signals such as LL-37, β defensin-3, and HMGB1, we find similar boosting activities of SIC (Figure 4 in Supplementary Material), suggesting that the protein is a general enhancer of inflammatory reactions once the immune system is alerted.

When testing different histone subclasses, we found that SIC binds exclusively to histone H3 and H4, which shows a specificity between the cationic histone subclasses. Our previous results with TLR antagonists demonstrated that extracellular histones alone can induce cytokine production through TLR4 on monocytes and macrophages (16). Noteworthy, SIC/histone complexes also induce IL-6 production through TLR4, suggesting that it activates the receptor using the same molecular mechanisms. In the same study, the cytokine-inducing residues of histone H4 was mapped to amino acids 35–54 (IRR20) (16). This binding site is different from the SIC-binding region that we have mapped to amino acids 1–20 (MSG20) of the N-terminal region of histone H4. These findings suggest a plausible explanation for how histone H4 can interact with SIC and simultaneously activate TLR4.

Since the cationic N-terminal part of histone H4 is responsible for the binding to SIC, the N-terminal parts of all histone subclasses were compared. The segments spanning the first 20 amino acids of histones H4 and H4 have both a net charge of +7, mediated by three arginine residues and four lysine residues. The corresponding sequences in H1 and H2B have one or no lysine residue, respectively, and a net charge of +4 (Table 3). Arginine is known to be cytotoxic to plasma membranes, and this may be an evolutionary reason as to why SIC has acquired affinity for H3 and H4 specifically. Though histone H2A has the same distribution of arginine and lysine residues and also a net charge of +7, it fails to interact with SIC. This finding may also indicate that structural elements are important for the interaction of SIC with histones. However, more experimental support is needed to draw a conclusive explanation for our results.

**Table 3:** Alignment of the N-terminal of all histone subclasses.

Protein	N-terminal sequence	Net charge	Arginines (R)	Lysines (K)
Histone H1	MTENSTSAPAAKPKRAKASK	+ 4	1	4
Histone H2A	MSGRGKQGGKARAKAKTRSS	+ 7	3	4
Histone H2B	MPEPAKSAPAPKKGSKKAVT	+ 4	0	5
Histone H3.1	MARTKQTARKSTGGKAPRKQ	+ 7	3	4
Histone H4	MSGRGKGGKGLGKGGAKRHR	+ 7	3	4

*The first 20 amino acids of each histone are depicted below, together with their net charge, and their arginine- and lysine content*.

In order to cause inflammation SIC has to interact with host proteins. This is in contrast to bacterial virulence factors which exert their pathological activity by interacting directly with their target receptor or cell. However, there are apart from SIC also other important virulence determinants using a similar approach. For example, LPS is using LPS-binding protein, a soluble acute-phase protein, to elicit immune responses through TLR4 (39). Streptococcal M-protein activates neutrophils by forming complexes with fibrinogen, while M-protein-induced TLR2 activation does not require fibrinogen (40). *S. pyogenes* and *Staphyloccocus aureus* release streptokinase and staphylokinase, respectively, two enzymes that can activate plasminogen and help the bacteria to disseminate from the site of infection (41) streptokinase (42). These examples show the sophisticated adaption of different pathogens to modulate the immune response and cause pathologic conditions.

To summarize, our *in vitro* data reveal that SIC/histone complexes elicit a broad upregulation of multiple pro-inflammatory cytokines, and it seems reasonable to speculate that SIC/histone complexes are formed *in vivo* as well. This leads to a cascade of events that amplifies inflammation and worsens the outcome of the infection, although the complex formation *in vivo* has to be further studied. Our study therefore shows that SIC has a Janus-faced function as it, on one hand, dampens the host reaction to infection, but on the other hand by its interaction with danger signals trigger massive inflammatory reactions.

## Ethics Statement

The study was approved by the Institutional review board (IRB) at the Lund University Hospital (Protocol no. 790/2005).

## Author Contributions

JW, LB, and HH conceived and designed the study. MM performed and analyzed the experiments in Figure 2. BC, JS, AN, and AN-T designed, performed, and analyzed colocalization experiments. JW designed, performed, and analyzed all remaining experiments, including Figures 1, 3, 4 and 5. I-MF purified SIC. MM and OH collected biopsies from patients with NSTI. I-MF and LB provided with intellectual content. JW and HH wrote the manuscript. All authors reviewed the results and approved the final version of the manuscript.

## Conflict of Interest Statement

The authors declare that the research was conducted in the absence of any commercial or financial relationships that could be construed as a potential conflict of interest.

## Abbreviations

GAS, group A streptococci; NSTI, necrotizing soft-tissue infection; AMPs, antimicrobial peptides; protein SIC, streptococcal inhibitor of complement; histones, calf thymus histones; SN, supernatant; CFUs, colony-forming units; HMGB1, high-mobility group box 1; HBP, heparin-binding protein; PBS-T, PBS containing 0.05% (v/v) Tween-20; PBS-G, PBS containing 5 mM glucose; ACIA, acquired computerized image analysis; TH, Todd–Hewitt; mga, multiple gene regulator of GAS.
