# Supplementary material for: Protein SIC Secreted from Streptococcus pyogenes Forms Complexes with Extracellular Histones That Boost Cytokine Production
Source: Front Immunol. 2018 Feb 22;9:236. doi: 10.3389/fimmu.2018.00236 (PMC5827136; doi:10.3389/fimmu.2018.00236)
Supplement: Supplementary file 1 [file data_sheet_1.docx]

Supplementary Material

**Protein SIC Secreted from *Streptococcus pyogenes* Forms Complexes with Extracellular Histones that Boost Cytokine Production**

**Johannes Westman^*1^, Bhavya Chakrakodi^2^, Johanna Snäll^2^, Matthias Mörgelin^3^, Martin Bruun Madsen^4^, Ole Hyldegaard^5^, Ariane Neumann^3^, Inga-Maria Frick^3^, Anna Norrby-Teglund^2^, Lars Björck^3^, Heiko Herwald^3^**

*** Correspondence:** Johannes Westman: Johannes.Westman@gmail.com

**Material and Methods**

**Negative staining and transmission electron microscopy**−Binding between SIC and histone H4 was visualized using negative staining and electron microscopy as previously described (30). Histone H4 (10 nm) and SIC (5 nm) were conjugated with colloidal gold particles according to routine protocols (31). Conjugates were incubated with each other for 30 min at RT and negatively stained with 0.75% uranyl formate. Specimens were examined in a Philips/FEI CM100 BioTwin transmission electron microscope at a X100,000 magnification.

**Multiplex cytokine quantification assay**−The cytokine analysis was performed using a Bio-Plex 200 instrument and a Bio-Plex Pro Human Cytokine 27-Plex Assay (both from Bio-Rad Laboratories, Berkeley, CA, USA) measuring FGF basic, Eotaxin, G-CSF, GM-CSF, IFN-γ, IL-1β, IL-1ra, IL-2, IL-4, IL-5, IL-6, IL-7, IL-8, IL-9, IL-10, IL-12, IL-13, IL-15, IL-17, CXCL10, CCL2, CCL3, CCL4, PDGF-bb, RANTES, TNF-α and VEGF. The cytokine panel was designed to provide a measure of cytokines, chemokines and growth factors. All samples, standards and controls were run in duplicates and data was acquired using Bio-Plex Manager Software (version 6.1).

**Immunostaining of tissue biopsies***−*Snap-frozen tissue biopsies from healthy controls or from patients with NSTI caused by *emm*1 or *emm*3 *S. pyogenes*strains were analyzed. The biopsies were cryosectioned to 8 μm and fixed in ice-cold acetone or 2% formaldehyde for immunofluorescent or immunohistochemical stainings, resp. Stainings were performed on consecutive sections as described previously (32,33). The following antibodies were used: mouse monoclonal anti-histone H4 (Abcam, Cambridge, UK), rabbit anti-SIC serum, goat polyclonal anti-Lancefield group A carbohydrate (Abcam, Cambridge, UK), and rabbit polyclonal antibodies against human HMGB1 (Abcam, Cambridge, UK) and human IL-8/NAP-1 (Invitrogen, Waltham, MA, USA). Biotinylated secondary antibodies included rabbit-anti-goat IgG and goat-anti-rabbit IgG (both from Vector Laboratories, Burlingame, CA, USA). For fluorescence stainings, Alexa 546 conjugated donkey anti-rabbit IgG and Alexa 488 conjugated donkey anti-mouse IgG (both from Molecular Probes, Eugene, OR, USA) were used. Slides were mounted using DAPI supplemented mounting media (Molecular Probes, Eugene, OR, USA). Single stainings were performed to assure specificity of staining patterns. For image evaluation, a Nikon A1R confocal microscope was used (Nikon Instruments, Amstelveen, the Netherlands).

**SUPPLEMENTAL FIGURES**

**SUPPLEMENTAL FIGURE 1. Original SDS-PAGE (A) and Western blot (B) for Figure 1.**


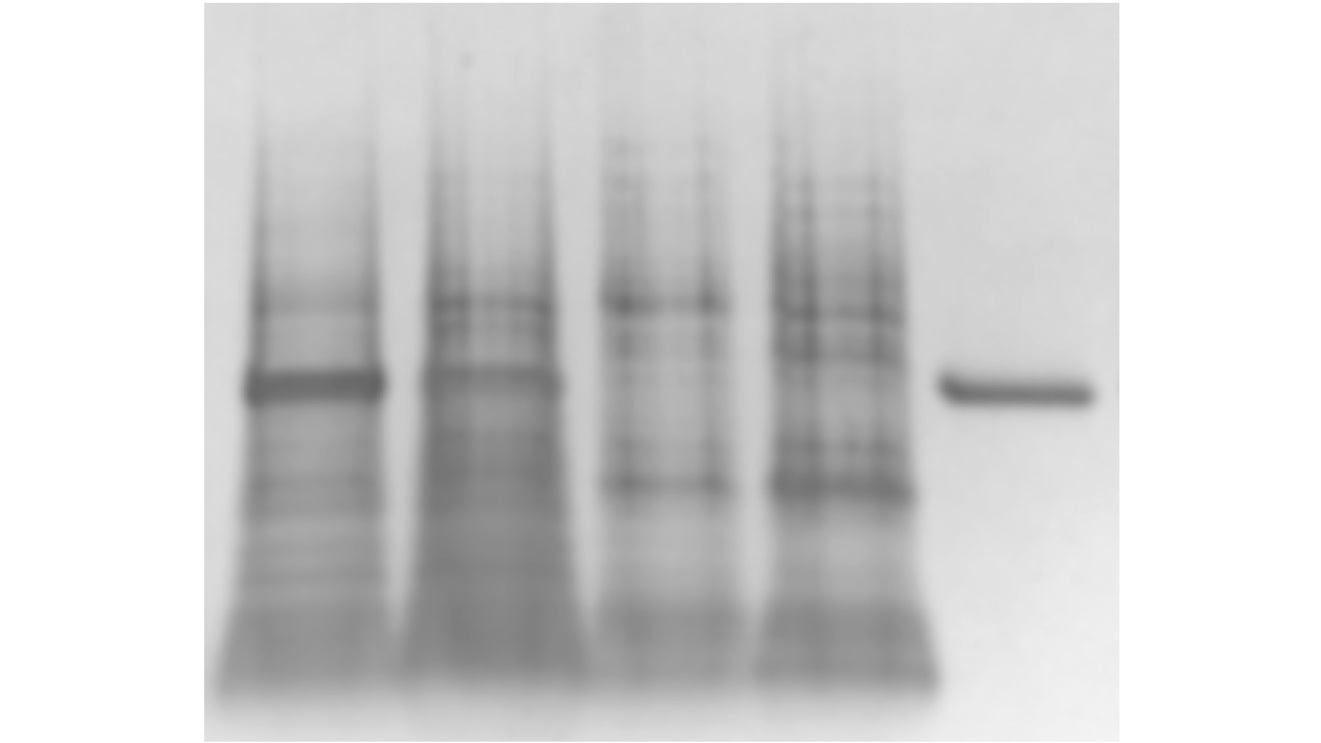
A

B **
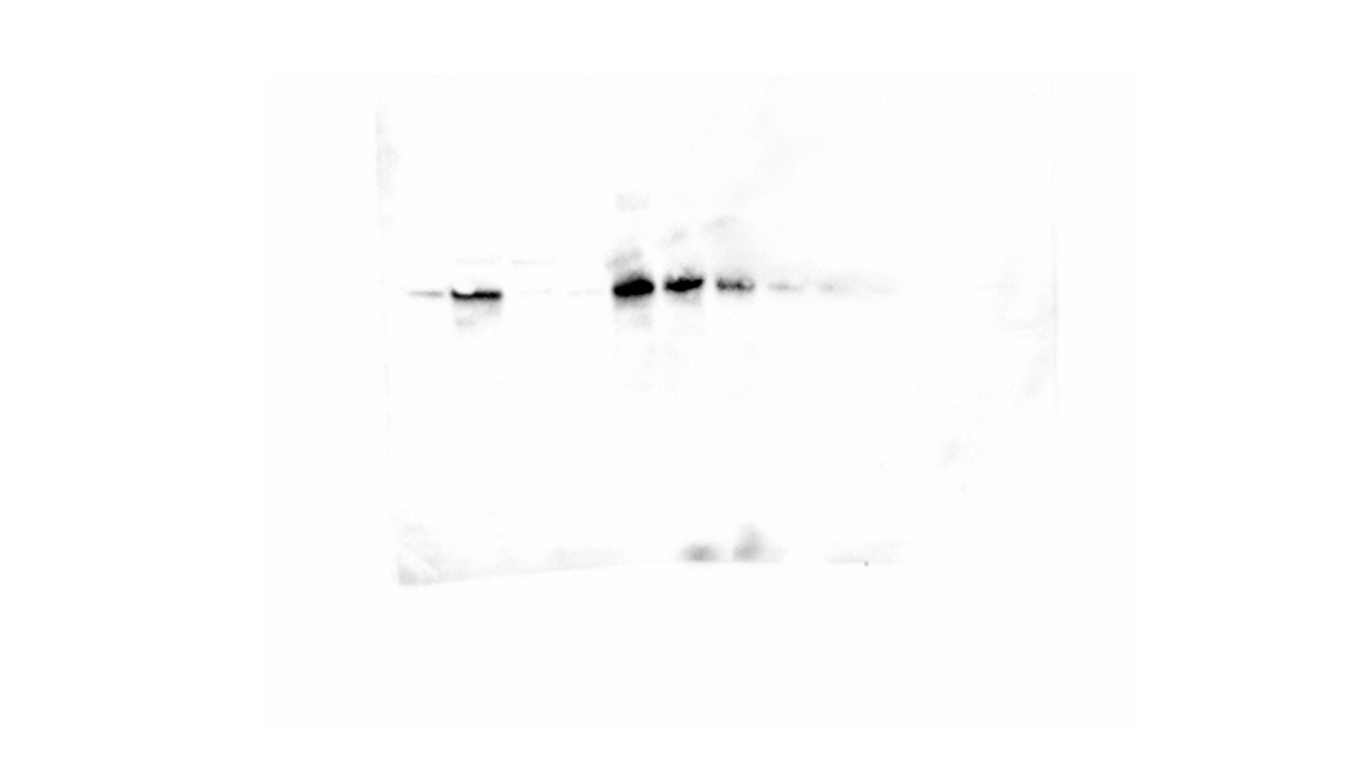
**


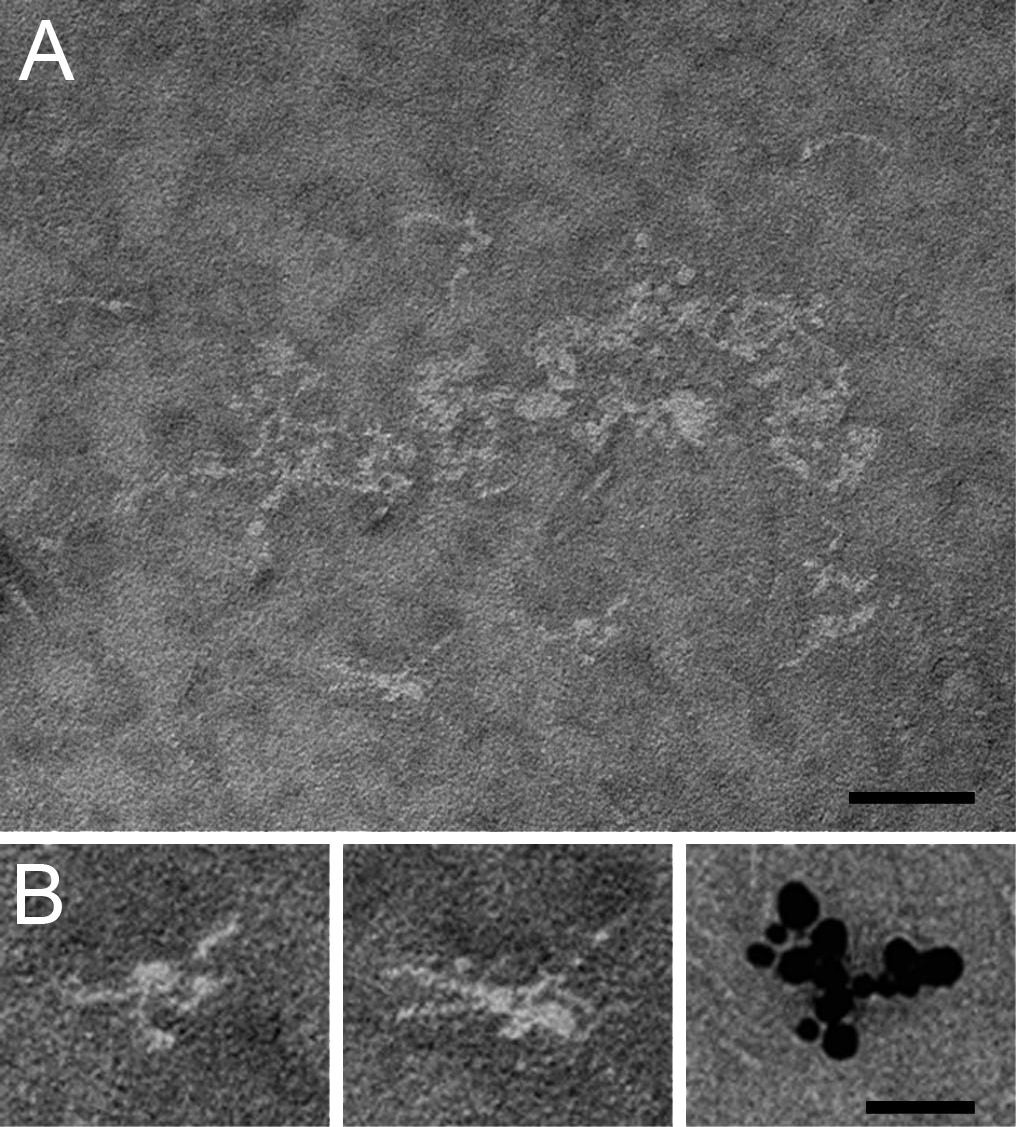


**SUPPLEMENTAL FIGURE 2.** Negative staining electron microscopy of SIC in complex with histone H4. Recombinant histone H4 was added to purified SIC. The formed aggregates were visualized using negative staining electron microscopy. (**A**) An overview of SIC/histone H4 complexes and larger aggregates is shown. Scale bar 50 nm. (**B**) Oligomers of aggregated SIC (5 nm gold particles) and histone H4 (10 nm gold particles) are depicted. Scale bar 25 nm.

**
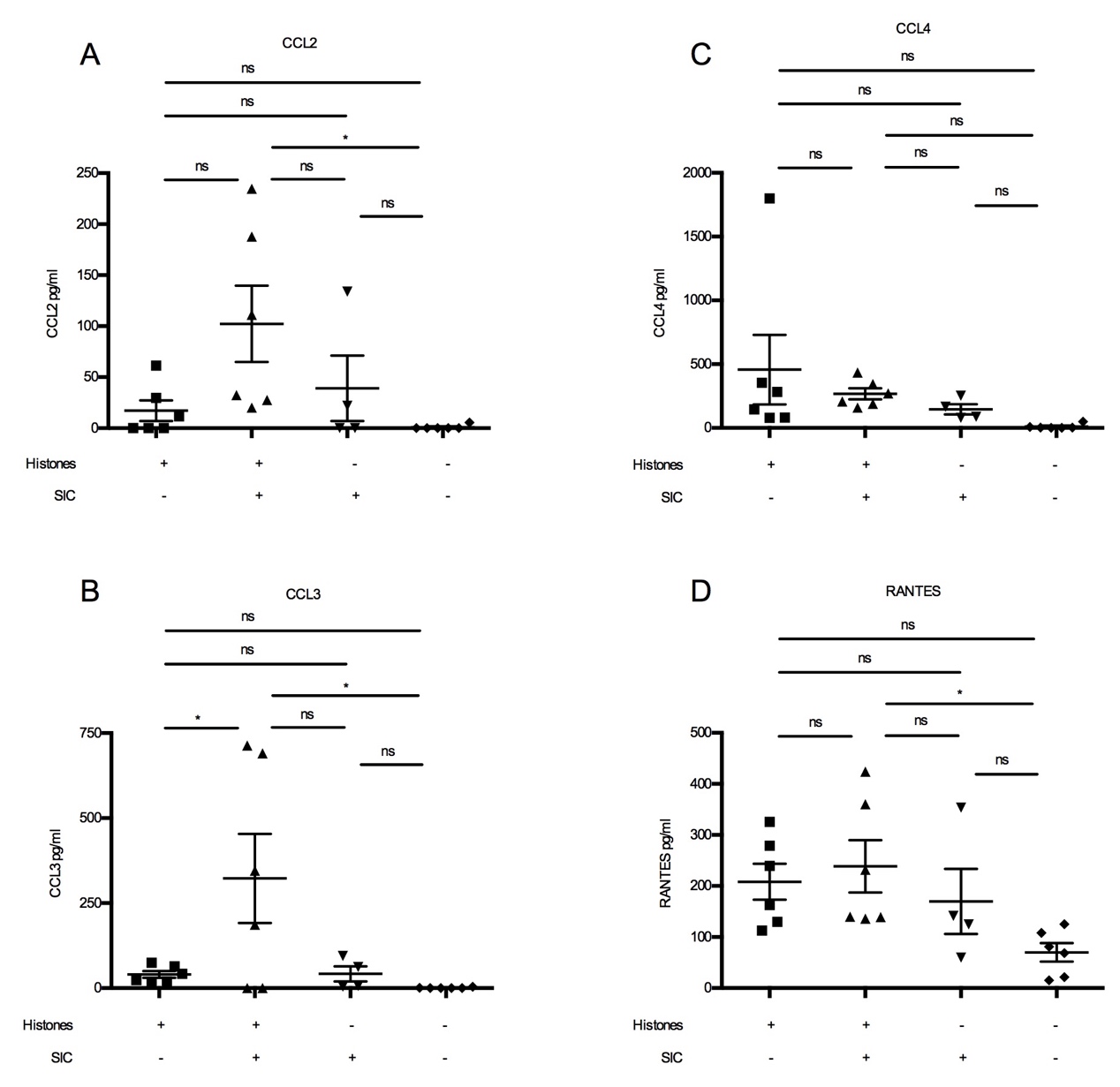
SUPPLEMENTAL FIGURE 3.** Cytokines and chemokines not induced by histones, SIC or the combination of the two proteins. Heparinized blood diluted 1:1 in PBS was stimulated with either histones (n=6), histones with SIC (n=6), SIC alone (n=4) or PBS (n=6) for 7 h at 37^o^C. Plasma SNs were analyzed for a panel of cytokines, chemokines and growth factors including CCL2 (**A**), CCL3 (**B**), CCL4 (**C**) and RANTES (**D**) using a Bio-Plex cytokine quantification assay. Data show mean +/- SEM (One-way ANOVA, Tukey’s test). All samples were measured in duplicates. P-values were determined to ≤ 0.05 (*), ≤ 0.01 (**) and ≤ 0.001 (***).

**
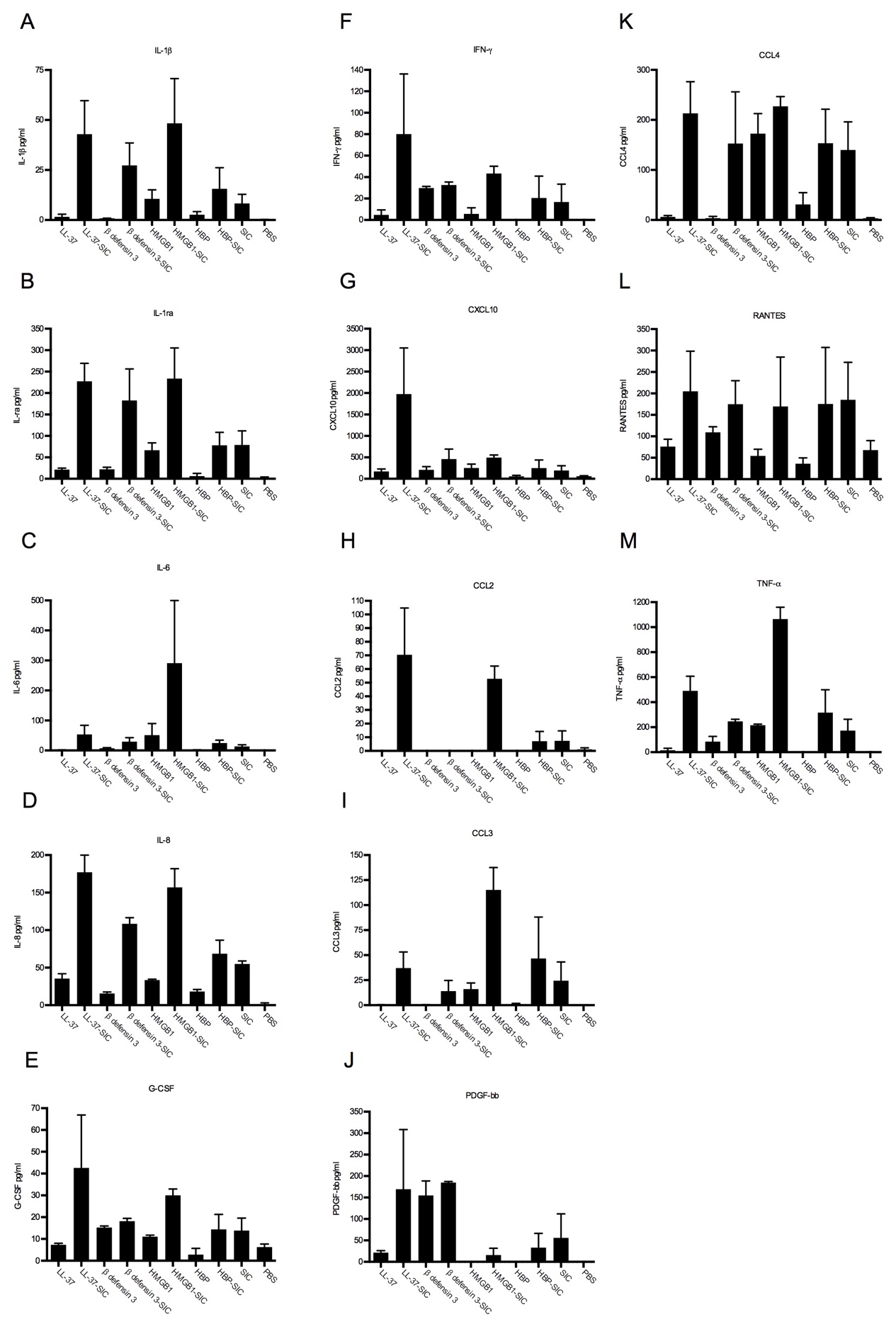
SUPPLEMENTAL FIGURE 4.** SIC/AMP interactions induce a broad range of cytokines and chemokines. Heparinized blood diluted 1:1 in PBS was stimulated with different AMPs (LL-37, β defensin-3, HMGB1, HBP, n=3), AMPs with SIC (n=3), SIC alone (n=3) or PBS (n=5) for 7 h at 37^o^C. Plasma SNs were analyzed for a panel of cytokines, chemokines and growth factors including IL-1β (A), IL-1ra (B), IL-6 (C), IL-8 (D), G-CSF (E), IFN-γ (F), CXCL10 (G), CCL2 (H), CCL3 (I), PDGF-bb (J), CCL4 (K), RANTES (L) and TNF-α (M) using a Bio-Plex cytokine quantification assay. Data show mean +/- SEM. All samples were measured in duplicates.

**
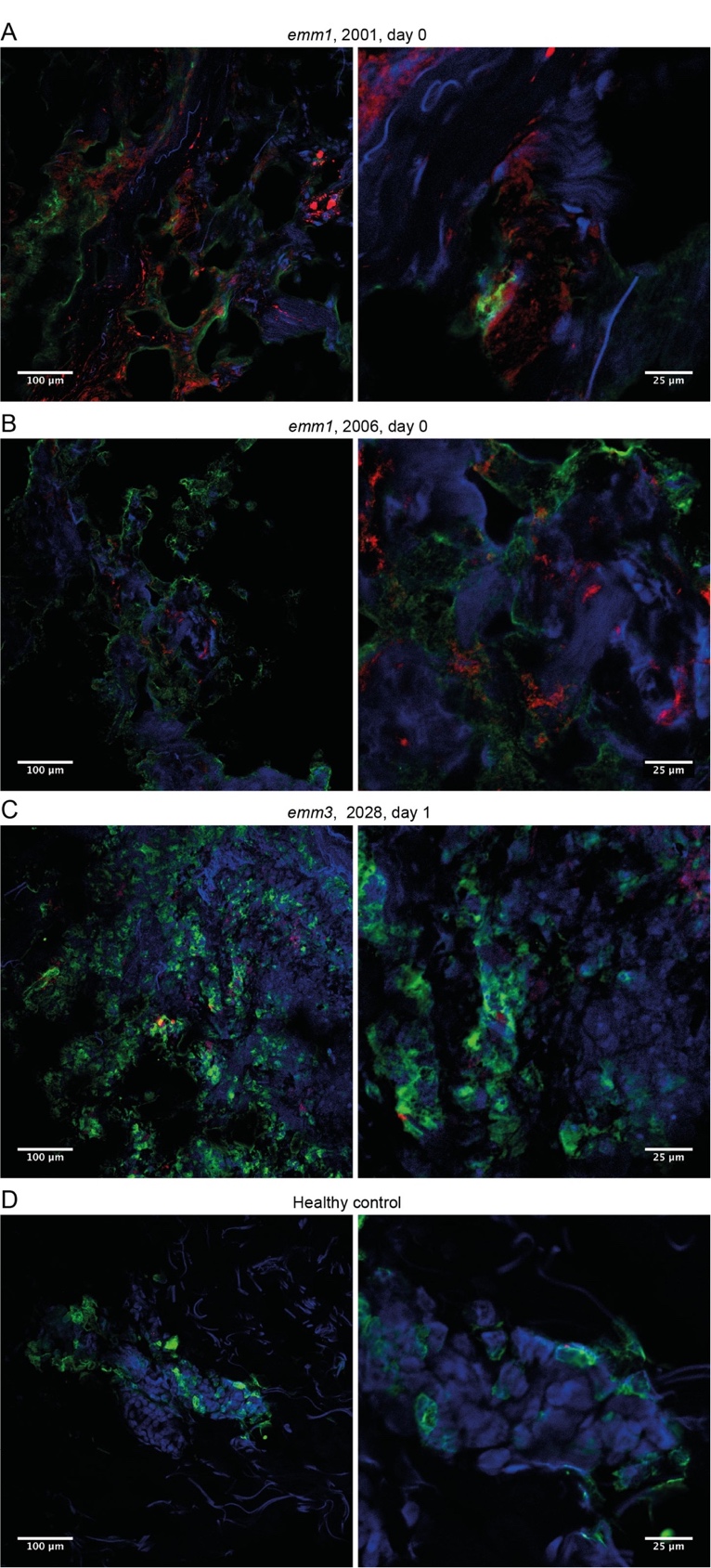
**

**SUPPLEMENTAL FIGURE 5.** SIC and histone H4 expression in patients with NSTI. Snap-frozen biopsies from patients with infections caused by *emm1* (**A** and **B**) or *emm3* (**C**) *S. pyogenes* strains were analyzed for SIC (red) and histone H4 (green) expression. DNA is stained with DAPI (blue). A biopsy from a healthy donor was used as a control (**D**). Scale bar 100 μm *(left panel)*, 25 μm (*right panel)*.
